# Adverse events and clinical risk factors during intrahospital transport of critically ill neonates: a prospective pilot study at a tertiary center in Vietnam

**DOI:** 10.3389/fped.2026.1850618

**Published:** 2026-06-05

**Authors:** Ho Tan Thanh Binh, Ho Truong Hong Hanh, Phi Duong Nguyen, Dinh Quang Truong

**Affiliations:** City Children’s Hospital, Ho Chi Minh City, Vietnam

**Keywords:** adverse events, Bayesian model averaging, hypothermia, intrahospital transport, neonatal transport, NICU safety, SpO_2_/FiO_2_ ratio, vasoactive-inotropic score

## Abstract

**Background:**

Intrahospital transport is often unavoidable in neonatal intensive care, but it may expose critically ill neonates to physiological instability and adverse events. Data from low- and middle-income settings remain limited, and differing hypothermia thresholds across studies may underestimate the true burden of transport-related harm.

**Methods:**

We conducted a prospective observational pilot study of intrahospital transport episodes from the neonatal intensive care unit (NICU) at a tertiary pediatric center in Ho Chi Minh City, Vietnam, between May and July 2024. Each transport episode was analyzed as a separate event. The primary outcome was unsafe transport, defined as at least one adverse event—including hypothermia <36.5°C, respiratory deterioration, hemodynamic instability, or device-related events—occurring during transport or within 24 h of NICU readmission. The secondary outcome excluded mild hypothermia and included only moderate hypothermia (32°C–35.9°C) and other clinically significant events.

**Results:**

Of the 172 screened transport events, 138 involving 96 neonates were included. Under the primary definition, 71 transport events (51.4%) were unsafe, generating 100 adverse events; 89% occurred during the return leg or post-return period. Hypothermia was the most frequent event (74.0%): 40 mild (36.0°C–36.4°C) and 34 moderate (32°C–35.9°C). Under the secondary definition, 44 transport events (31.9%) were unsafe, with 60 adverse events. Transport was significantly associated with increased FiO_2_ (*p* = 0.008) and reduced SpO_2_/FiO_2_ ratio (*p* = 0.020). A surgical indication was the strongest independent predictor: moderate Bayesian model averaging evidence for the primary outcome [posterior inclusion probability (PIP) 0.616] and strong evidence for the secondary outcome (PIP 0.880; odds ratio 4.95, 95% credible interval 1.43–17.2). Moderate hypothermia was independently associated with support escalation (41.2% vs. 18.6%, *p* = 0.018), whereas mild hypothermia was not. The vasoactive-inotropic score showed negligible predictive value, equivalent to vasoactive drug count.

**Conclusions:**

Intrahospital transport of critically ill neonates is frequently associated with adverse events, predominantly hypothermia occurring on the return leg. A surgical indication—particularly for esophageal atresia, gastroschisis, or urgent procedures — is the dominant independent risk factor. Improved thermal protection (including routine incubator use for surgical cases), standardized postreturn temperature assessment, and enhanced preparation for surgical transport are the highest-priority evidence-based interventions.

## 1 Introduction

Critically ill neonates frequently require intrahospital transport for specialized imaging and surgical procedures that cannot be performed at the bedside (1–5). Although such transport events occur within a single institution over relatively short distances, they expose fragile infants to environmental changes, interruptions in monitoring, and manipulation of life-sustaining equipment—each of which may precipitate physiological instability (4–7). Complication rates during intrahospital transport range from 25% to 70% in pediatric and adult intensive care units, with comparable rates reported in neonatal cohorts from high-income countries (1, 2, 4, 6–8).

Data specifically addressing intrahospital neonatal transport in low- and middle-income countries (LMICs) remain scarce. Studies from Brazil and Switzerland have reported adverse event rates of 25%–30%, identifying hypothermia, respiratory deterioration, and hemodynamic instability as the most frequent complications (1, 2). A critical methodological issue is the variability in the hypothermia threshold: some studies use <36.0°C, while the WHO neonatal standard is <36.5°C (1, 3, 9). This discrepancy makes cross-study comparisons unreliable and risks the systematic underestimation of the thermal burden of transport since mild hypothermia (36.0°C–36.4°C) is not clinically benign—it can exacerbate metabolic acidosis, impair surfactant function in preterm infants, and increase vulnerability to intraventricular hemorrhage (10–12).

The vasoactive-inotropic score (VIS), integrating the number and potency of vasoactive agents, is an established composite severity measure in neonatal and pediatric critical care (13). Whether the VIS outperforms a simple vasoactive drug count as a transport risk predictor has not been previously evaluated (14, 15).

Our institution is a tertiary pediatric referral center in southern Vietnam with a high volume of neonatal surgical and cardiac cases, many of which require repeated transfers between the neonatal intensive care unit (NICU), operating rooms, and imaging departments. Despite the frequency of these transfers, there has been no systematic evaluation of their safety in this context.

This pilot study had four objectives: (1) to determine the incidence and spectrum of adverse events using two complementary outcome definitions—a primary outcome based on the WHO hypothermia standard (<36.5°C) capturing the full clinical burden and a secondary outcome excluding mild hypothermia to isolate clinically significant instability and yield a stronger risk model; (2) to characterize transport-associated physiological changes; (3) to identify independent risk factors, including VIS, using a Bayesian information criterion (BIC)-based Bayesian model averaging (BMA) across all predictor combinations; and (4) to provide stratified outcome analyses by hypothermia severity to inform the clinical relevance of the dual-threshold approach.

## 2 Methods

### 2.1 Study design and setting

This was a single-center, prospective, observational pilot study conducted in the NICU of a tertiary pediatric hospital in Ho Chi Minh City, Vietnam, between May and July 2024. The NICU admits high-risk neonates from within the hospital and by referral, with a substantial proportion having congenital anomalies requiring surgical or interventional management. Each transport event was analyzed as a distinct observation. All transport events were handled by dedicated NICU teams using standard equipment and protocols. No patient required extracorporeal membrane oxygenation (ECMO) during transport.

### 2.2 Study population

All intrahospital transport events originating from the NICU were screened prospectively. The inclusion criteria were as follows: (1) age <29 days for neonates ≥32 weeks gestational age (GA), or ≤40 weeks postmenstrual age for those <32 weeks GA; (2) admission to the NICU; (3) receipt of invasive or non-invasive respiratory support, hemodynamic support (vasoactive drugs), or parenteral nutrition at the time of transport; and (4) transport for imaging, surgery, or other procedures outside the NICU. Transport was excluded if the infant required no cardiorespiratory support on full enteral feeds, had incomplete data, or did not return to the NICU.

### 2.3 Outcome definitions

#### 2.3.1 Primary outcome

Unsafe transport was defined as at least one adverse event recorded during transport or within 24 h of NICU readmission. Adverse events comprised the following: (1) hypothermia—axillary temperature <36.5°C per WHO neonatal standards, (9) classified as mild (36.0°C–36.4°C) or moderate (32°C–35.9°C); (2) hemodynamic instability (mean arterial pressure below gestational age-appropriate thresholds, or bradycardia, tachycardia, or the requirement for vasoactive escalation); (3) respiratory deterioration (SpO_2_ < 90% preterm or <92% term, or the requirement for ventilatory escalation); and (4) device-related events (dislodged lines, tubes, or equipment failure).

#### 2.3.2 Secondary outcome

The secondary outcome was the same as the primary outcome, except that mild hypothermia (36.0°C–36.4°C) was excluded. An unsafe secondary transport was defined as experiencing at least one of the following: moderate hypothermia (32°C–35.9°C), hemodynamic instability, respiratory deterioration, or a device-related event. This stricter definition concentrates on instability requiring clinical intervention and, as demonstrated by BMA, yields superior risk model performance.

Adverse events in the 24-h post-return period were interpreted in relation to their temporal proximity to transport and with appropriate caution, as postoperative instability may occur independently*.*

### 2.4 Data collection

Standardized prospective forms captured the following: demographics and perinatal characteristics, primary diagnosis, transport indication, transport vehicle (bed/warmer/incubator), total duration outside the NICU, team composition, respiratory support level at each trip leg, VIS and vasoactive drug count (pretransport), nutritional support, vascular access, and surgical procedure type. Adverse events and their precise timing were documented by the transport team.

Axillary temperature was measured at standardized time points—immediately before departure and upon returning to the NICU—using a validated digital electronic thermometer, performed by a transport team member. Ambient temperatures in operating theaters and imaging suites were not systematically recorded; this is acknowledged as a limitation.

The SpO_2_/FiO_2_ ratio was calculated from pulse oximetry and FiO_2_ values recorded immediately before transport. VIS = dopamine (μg/kg/min) + dobutamine (μg/kg/min) + 100 × epinephrine (μg/kg/min) + 100 × norepinephrine (μg/kg/min) + 10 × milrinone (μg/kg/min) + 10,000 × vasopressin (units/kg/min). VIS was analyzed as a continuous variable in BMA and described categorically as VIS = 0, 1–9, or ≥10.

### 2.5 Statistical analysis

Continuous variables were presented as means ± SD or medians with IQR; categorical variables were presented as counts and percentages. Pre–post physiological comparisons used paired Wilcoxon signed-rank tests. Group comparisons used Mann–Whitney *U* or Fisher's exact/chi-squared tests as appropriate. The SpO_2_/FiO_2_ ratio predictive performance for each outcome was assessed by performing an ROC analysis [area under the ROC curve (AUC) with 95% CI, Youden optimal threshold, sensitivity, specificity, and odds ratio at the threshold].

Risk factors for unsafe transport were first explored in univariate analyses and then assessed employing BMA logistic regression across all six prespecified predictors (gestational age, transport duration, invasive mechanical ventilation (MV), pretransport VIS, surgical indication, and age at transport) using a uniform prior over all 64 candidate models. Coefficient priors were represented by the BIC approximation, corresponding to a unit-information prior. Posterior model probabilities were normalized from BIC-derived likelihood weights. Posterior inclusion probabilities (PIPs) represent the summed posterior weight of models containing each predictor. BMA ORs represent the probability-weighted average maximum likelihood estimate (MLE) across models. Evidence thresholds: PIP ≥ 0.75 strong; 0.50–0.74 moderate; 0.25–0.49 weak; <0.25 negligible.

BMA was performed at the transport-event level. To assess the magnitude of possible within-infant dependency, we found that 28 of the 96 infants (29.2%) contributed more than one transport; the correlation between successive outcomes within the same infant was low (*r* = 0.169, *p* = 0.490), consistent with independent event-level analysis. Each transport event represents a clinically distinct episode with different indications, duration, physiological starting conditions, and team composition. As a prespecified sensitivity analysis, the BMA was repeated, retaining only the first transport event per patient (*n* = 96). In that analysis, surgical indication remained the strongest predictor of PIP directionally for both outcomes (primary PIP 0.520, secondary PIP 0.636); attenuation of PIPs reflected reduced statistical power from the smaller event set rather than inconsistency in the direction of evidence. A stratified outcome analysis by hypothermia severity was performed using Fisher's exact test. All analyses were performed in *R*; the BIC-BMA procedure is equivalent to the *R* BMA package.

### 2.6 Ethics

This study was approved by the City Children's Hospital ethics committee and conducted in accordance with local legislation and institutional requirements. Written informed consent for participation was provided by the participants’ legal guardians/next of kin. Data were deidentified. The study was reported in accordance with the STROBE checklist.

## 3 Results

### 3.1 Patient and transport characteristics

Of the 172 screened transport events, 138 involving 96 neonates met the inclusion criteria. The median gestational age was 37.0 weeks (IQR 35.3–38.0); 52 (37.7%) were preterm (<37 weeks). The median birth weight was 2,725 g (IQR 2,200–3,100); 31.9% involved infants <2,500 g. Male neonates accounted for 51.4% of the events; the median postnatal age at transport was 11 days (IQR 5–20); 19.6% of the transport events occurred within the first 72 h of life. Congenital surgical anomalies were the predominant diagnosis (Table 1).

**Table 1:** Primary diagnosis categories (*N* = 96 patients).

Primary diagnosis category	*n* (%)
Congenital surgical conditions (abdominal wall, GI, Hirschsprung disease, etc.)	55 (59.8)
Congenital heart disease	13 (14.1)
Neurological disorders (HIE, seizures, hydrocephalus)	10 (10.9)
Infectious diseases including sepsis	9 (9.8)
Prematurity-related respiratory disorders	8 (8.7)
Other (airway anomalies, cystic lesions, etc.)	7 (7.6)

Diagnoses not mutually exclusive; six patients (6.3%) had coexisting conditions spanning two diagnostic categories. Percentages are calculated with *N* = 96 as the denominator and summed to >100%.

Surgery was the leading transport indication (72/138, 52.2%). On the outward trip, invasive mechanical ventilation was required in 42.8% of events; any vasoactive support (VIS > 0) was present in 23.9% of transport events: 12.3% had VIS 1–9 and 11.6% had VIS ≥10; the median VIS was 0 (IQR 0–0). The median transport duration was 93 min (IQR 54–168). The majority of transport events (94.2%) involved at least one physician and one nurse; a transport incubator was used for only 4.3% of outward trips (Table 2).

**Table 2:** Transport event characteristics (*N* = 138).

Variable	*n* (%) or median (IQR)
Transport indication—surgery	72 (52.2)
Transport indication—diagnostic imaging/other	66 (47.8)
Invasive MV (outward trip)	59 (42.8)
Non-invasive ventilation/CPAP (outward)	28 (20.3)
Supplemental oxygen only (outward)	12 (8.7)
Room air (outward)	39 (28.3)
Any vasoactive support (VIS > 0)	33 (23.9)
VIS 1–9	17 (12.3)
VIS ≥10	16 (11.6)
VIS pretransport, median (IQR)	0 (0–0)
Physician + nurse team	130 (94.2)
Transport incubator (outward)	6 (4.3)
Time outside NICU (min), median (IQR)	93 (54–168)

CPAP, continuous positive airway pressure.

### 3.2 Adverse events—primary and secondary outcomes

Of the 71 unsafe transport events, 49 (69.0%) had one AE, 15 (21.1%) had two AEs, and 7 (9.9%) had three AEs, generating a total of 100 AEs. Of these 100 AEs, 11 (11.0%) occurred during the outward trip and 89 (89.0%) occurred during the return leg or post-return period.

Hypothermia (<36.5°C) accounted for 74 of the 100 adverse events (74.0%): 40 were mild (36.0°C–36.4°C), and 34 were moderate (32°C–35.9°C); no severe hypothermia was recorded. Notably, six of the 40 mild episodes of hypothermia occurred during the outward trip and the remaining 34 during the return leg, while all 34 moderate episodes of hypothermia occurred during the return leg. Non-hypothermia events accounted for 26 (26.0%): respiratory deterioration (10, 10.0%), hemodynamic instability (12, 12.0%), and device-related problems (4, 4.0%).

Under the WHO Harm Classification, the majority of events were categorized as “no harm” or “mild harm” (reversible temperature or oxygenation abnormalities); a smaller proportion was categorized as “moderate harm” (escalation of support without persistent disability). No transport-related deaths occurred.

No adverse events were recorded before departure. Of the 71 unsafe transport events, 23 (32.4%) first occurred during transport, whereas 48 (67.6%) first manifested upon NICU readmission or within 6 h thereof. Under the secondary outcome definition, 44/138 (31.9%) were unsafe events, generating 60 adverse events (34 cases of moderate hypothermia + 26 cases of non-hypothermia; Table 3).

**Table 3:** Adverse events under primary and secondary outcome definitions.

Variable	Primary outcome (hypo <36.5°C) 100 events/71 transport events	Secondary outcome (hypo <36.0°C) 60 events/44 transport events
Hypothermia, total	74 (74.0%)	34 (56.7%)
Mild (36.0°C–36.4°C)	40 (40.0%)	— (excluded)
Moderate (32°C–35.9°C)	34 (34.0%)	34 (56.7%)
Non-hypothermia events	26 (26.0%)	26 (43.3%)
* *Respiratory deterioration	10 (10.0%)	10 (16.7%)
Hemodynamic instability	12 (12.0%)	12 (20.0%)
* *Device-related events	4 (4.0%)	4 (6.7%)
Total adverse events	100	60
Unsafe transport events	71/138 (51.4%)	44/138 (31.9%)
AE per unsafe transport (primary)		—
1 event	49 (69.0%)	—
2 events	15 (21.1%)	—
3 events	7 (9.9%)	—
Timing: AE episodes by trip leg		—
Outward trip	11 (11.0%)	—
Return/postreturn (≤ 6 h)	89 (89.0%)	—
Timing: first AE per unsafe transport		—
During the outward trip	11 (11.0%)	5 (8.3%)
During the return trip	20 (20.0%)	20 (33.3%)
At NICU readmission	62 (62.0%)	28 (46.7%)
Within 2 h after return	6 (6.0%)	6 (10.0%)
Within 2–6 h after return	1 (1.0%)	1 (1.7%)

AE, adverse event. Non-hypothermia counts confirmed from transport team records. Secondary AE: 34 moderate hypo + 26 non-hypo = 60.

### 3.3 Stratified outcome analysis by hypothermia severity

Moderate hypothermia (<36.0°C) was significantly associated with increased support escalation compared with no hypothermia (41.2% vs. 18.6%, *p* = 0.018), while mild hypothermia was not (17.6% vs. 18.6%, *p* = 0.87). In-hospital mortality did not differ across groups (*p* = 0.343), reflecting the high baseline disease severity of this cohort. These data support the dual-threshold approach: mild hypothermia is a prevention target, whereas moderate hypothermia is a clinically consequential harm associated with escalation of care (Table 4).

**Table 4:** Clinical consequences by hypothermia severity (*N* = 138).

Outcome	No hypothermia (*n* = 70), *n* (%)	Mild hypo 36.0°C–36.4°C (*n* = 34), *n* (%)	Moderate hypo 32°C–35.9°C (*n* = 34), *n* (%)
Any support escalation^a^	13 (18.6)	6 (17.6)	14 (41.2)*
In-hospital mortality	11 (15.7)	5 (14.7)	9 (26.5)
Persistent hypothermia at T6 h (< 36.5 °C)	3 (4.3)	0 (0.0)	2 (5.9)

a Support escalation: any increase in FiO_2_ > 10%, MV level, or VIS within 6 h of return. Fisher’s exact test.

* *p* = 0.018 vs. the no-hypothermia group. Overall mortality (25/138, 18.1%) reflects underlying disease severity. The mild vs. moderate comparison *p* = 0.061.

### 3.4 Physiological changes associated with transport

Paired Wilcoxon analyses comparing pretransport with 6-h post-transport values demonstrated significant changes in two oxygenation parameters (Table 5). FiO_2_ increased from 28.9% ± 11.0% to 32.3% ± 16.1% (*δ* + 3.34%, 95% CI 1.34–5.34; *p* = 0.008), and the SpO_2_/FiO_2_ ratio decreased from 361.8 ± 94.8 to 342.6 ± 105.7 (*δ* −19.1, 95% CI −32.6 to −5.7; *p* = 0.020). Heart rate (*p* = 0.777), SpO_2_ (*p* = 0.797), and axillary temperature (*p* = 0.493) did not change significantly, which is consistent with the temporal pattern in which hypothermia predominantly manifested during the return leg rather than uniformly throughout transport.

**Table 5:** Paired physiological changes before and 6 h after transport (*N* = 138).

Parameter	Pretransport (mean ± SD)	6 h Post-transport (mean ± SD)	*p*-Value
Heart rate (bpm)	145.4 ± 13.6	146.6 ± 12.8	0.777
SpO_2_ (%)	95.4 ± 2.5	95.4 ± 2.3	0.797
FiO_2_ (%)	28.9 ± 11.0	32.3 ± 16.1	0.008^a^
Axillary temperature (°C)	36.72 ± 0.32	36.74 ± 0.37	0.493
SpO_2_/FiO_2_ ratio	361.8 ± 94.8	342.6 ± 105.7	0.020^a^

a Statistically significant. Paired Wilcoxon signed-rank test. SpO_2_/FiO_2_ = SpO_2_ (%)/FiO_2_ (fraction). *n* = 138 complete pairs for all parameters.

**Figure 1:**
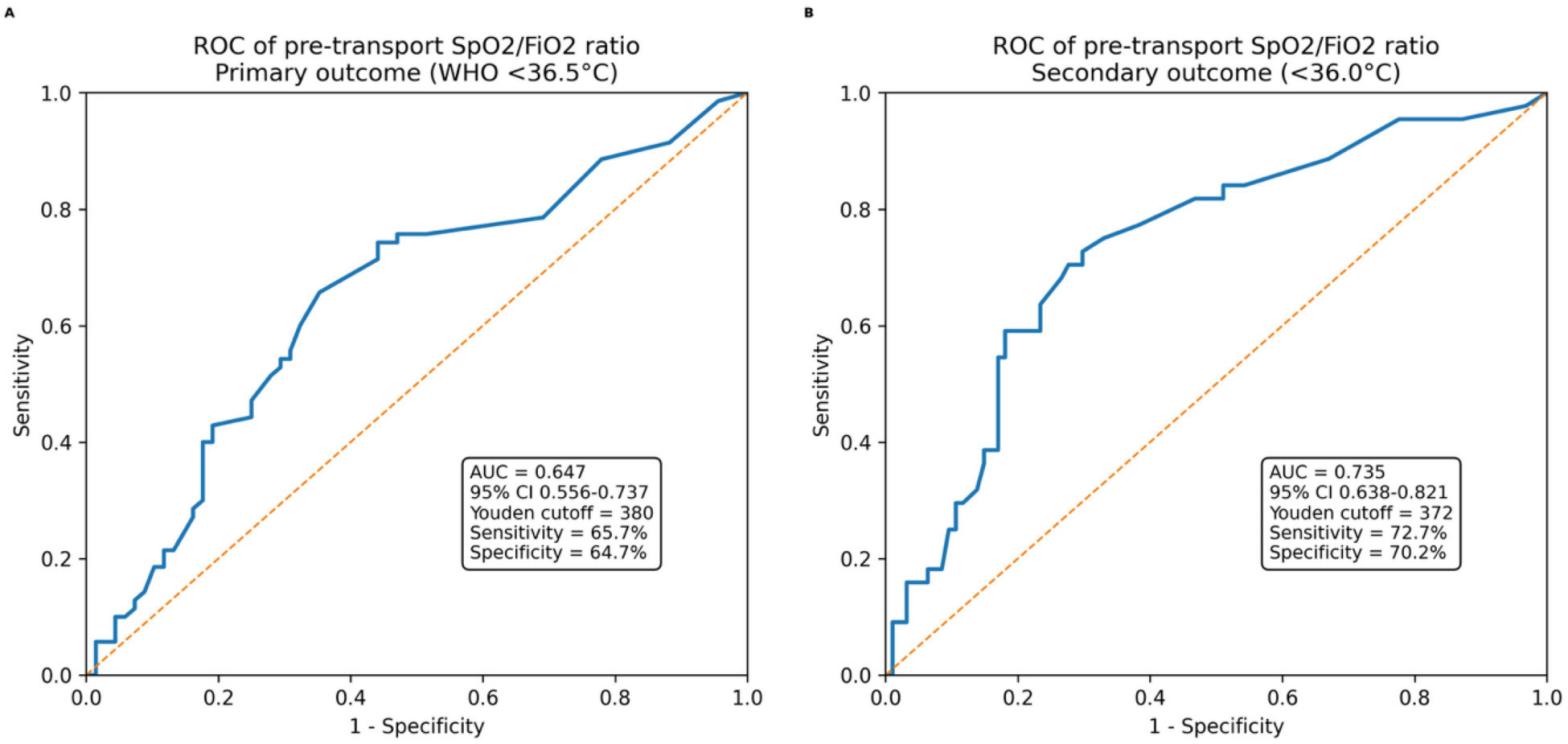
Receiver operating characteristic curves of the pretransport SpO_2_/FiO_2_ ratio for predicting adverse events. **(A)** Primary outcome defined using the WHO-based hypothermia threshold (<36.5°C). **(B)** Secondary outcome defined using the <36.0°C threshold. Youden-optimal threshold points are marked with filled circles; a reference diagonal is included. AUC, area under the ROC curve; OR, odds ratio. The SpO_2_/FiO_2_ ratio is calculated from pulse oximetry and FiO_2_ recorded immediately before transport. A 95% CI was estimated by bootstrapping (1,000 iterations).

### 3.5 Predictive performance of the pre-transport SpO_2_/FiO_2_ ratio

ROC analyses were performed separately for each outcome (Figure 1). For the primary outcome, the pretransport SpO_2_/FiO_2_ ratio showed moderate discriminative ability (AUC 0.644, 95% CI 0.56–0.73); the Youden index identified an optimal threshold of 392, with a sensitivity of 76.1%, a specificity of 53.7%, and an odds ratio (OR) of 3.4 for adverse events below this threshold. For the secondary outcome, the discriminative performance was meaningfully higher (AUC 0.735, 95% CI 0.65–0.82; threshold 372; sensitivity 72.7%, specificity 70.2%; OR 5.6). The improved secondary performance reflects the mechanistic link between oxygenation status and cardiorespiratory instability, unconfounded by mild hypothermia events not predicted by the SpO_2_/FiO_2_ ratio. The SpO_2_/FiO_2_ ratio is increasingly recognized as a reliable non-invasive surrogate for the PaO_2_/FiO_2_ ratio in pediatric acute lung injury (16–18); neonates with a SpO_2_/FiO_2_ ratio <372 likely represent a subgroup with significant underlying parenchymal lung disease or impaired gas exchange efficiency, making them more vulnerable to the physiological stressors of transport. Future studies should validate the predictive performance of a SpO_2_/FiO_2_ threshold of 372 for clinically significant transport-related adverse events in independent, larger neonatal cohorts, ideally stratified by surgical and non-surgical indications and incorporating validated illness severity scores as covariates.

### 3.6 Risk factors: Bayesian model averaging

Univariate analyses are summarized in Table 6. For the primary outcome, invasive MV (78.9% vs. 52.2%, *p* = 0.002), surgical indication (66.2% vs. 37.3%, *p* = 0.001), transport duration (median 120 vs. 72 min, *p* = 0.001), and pretransport SpO_2_/FiO_2_ ratio (median 320 vs. 448, *p* = 0.003) were significantly associated with unsafe transport. Pretransport VIS was highly right-skewed: median 0 (IQR 0–0; range 0–31); 76.1% of transports occurred without vasoactive support. Univariate VIS comparison did not reach significance (*p* = 0.070 primary, *p* = 0.086 secondary). VIS was nearly perfectly collinear with vasoactive drug count (Spearman *r* = 0.927, *p* < 0.001). For the secondary outcome, the signals were stronger: surgery (81.8% vs. 38.3%, *p* < 0.001), invasive MV (86.4% vs. 56.4%, *p* = 0.001), and transport duration (median 162 vs. 72 min, *p* < 0.001).

**Table 6:** Univariate analysis: safe vs. unsafe transport by outcome definition.

Variable	Primary safe (*n* = 67)	Primary unsafe (*n* = 71)	*p*	Secondary unsafe (*n* = 44)	*p*
GA (weeks), median (IQR)	37.0 (36–38)	37.0 (35–38)	0.901	37.0 (35–38)	0.533
Duration (min), median (IQR)	72 (45–150)	120 (84–186)	0.001*	162 (100–213)	<0.001*
Invasive MV, *n* (%)	35 (52.2)	56 (78.9)	0.002*	38 (86.4)	0.001*
Surgical indication, *n* (%)	25 (37.3)	47 (66.2)	0.001*	36 (81.8)	<0.001*
VIS pretransport, median (IQR)	0 (0–0)	0 (0–5)	0.070	0 (0–5)	0.086
SpO_2_/FiO_2_, median (IQR)	448 (317–457)	320 (271–392)	0.003*	—	<0.001*

GA, gestational age; MV, mechanical ventilation; VIS, vasoactive-inotropic score. Secondary safe *n* = 95.

* *p* < 0.05.

BMA logistic regression across 64 models was performed independently for each outcome (Table 7). For the primary outcome, surgical indication was the leading predictor [PIP 0.616, moderate evidence; BMA OR 1.96, 95% credible interval (CrI) 0.67–5.74], followed by invasive MV (PIP 0.490, weak). VIS achieved negligible evidence (PIP 0.083). For the secondary outcome, surgical indication reached strong BMA evidence (PIP 0.880; BMA OR 4.95, 95% CrI 1.43–17.2)—the only predictor to do so in either analysis. Transport duration showed weak evidence (PIP 0.316; BMA OR 1.002/min). All other predictors had negligible PIPs (<0.25). In the sensitivity analysis, replacing VIS with vasoactive drug count, all PIP values for the shared predictors were identical (± 0.001), and vasoactive count achieved a PIP = 0.080 vs. a PIP = 0.079 for VIS, confirming that VIS offers no additional predictive value over drug count in this cohort.

**Table 7:** Bayesian model averaging results (*N* = 138, 64 models).

Predictor	PIP (primary)	BMA OR (primary)	95% CrI (primary)	PIP (secondary)	BMA OR (secondary)	95% CrI (secondary)
Surgical indication	0.616	1.961	0.670–5.741	0.880^a^	4.954	1.428–17.189
Invasive MV	0.490	1.673	0.574–4.878	0.152	1.113	0.664–1.867
Age at transport (per day)	0.179	0.997	0.983–1.011	0.136	0.998	0.986–1.009
Transport duration (per min)	0.145	1.000	0.998–1.003	0.316	1.002	0.996–1.009
Gestational age (per week)	0.088	1.002	0.988–1.016	0.097	0.997	0.972–1.022
VIS pretransport (per unit)	0.083	0.999	0.993–1.006	0.079	1.000	0.998–1.003

a Strong evidence (PIP ≥ 0.75). Evidence thresholds: ≥0.75 strong; 0.50–0.74 moderate; 0.25–0.49 weak; <0.25 negligible. BIC-BMA; uniform model prior (1/64); Zellner unit information coefficient prior (g = n); 2⁶ = 64 models. Sensitivity analysis replacing VIS with vasoactive drug count: PIP=0.080 (count) vs. 0.079 (VIS). Yellow = primary clinical finding.

Neonates with congenital heart disease (CHD; 23/138 transport events, 16.7%) showed paradoxically lower primary unsafe rates (30.4% vs. 55.7%; *p* = 0.040) and secondary unsafe rates (13.0% vs. 35.7%; *p* = 0.048) despite higher VIS scores (VIS > 0: 39.1% vs. 20.9%). This may reflect shorter transport durations in CHD patients (median 60 vs. 96 min), with cardiac catheterization/stent being the only surgical subgroup with 0% adverse events or targeted preparation by the cardiac NICU team (Supplementary Table S2).

### 3.7 Surgical procedure breakdown

A detailed breakdown of the 72 surgical transport events by procedure type and urgency is provided in Supplementary Table S1. Esophageal atresia, gastroschisis, omphalocele, neurosurgical procedures, and patent ductus arteriosus (PDA) ligation had the highest primary unsafe rates (100%) among elective procedures. Urgent surgical cases (*n* = 8 events) had ≥80% primary and secondary unsafe rates. Cardiac catheterization/stenting was the only surgical subgroup with 0% adverse events, likely reflecting the dedicated preparation of the cardiac team. No patient required ECMO during transport.

### 3.8 Clinical outcomes

Among neonates with adverse events, 41% recovered spontaneously without escalation of therapy; 20% required increased respiratory support within 24 h, and 6.8% required additional vasoactive medications. The median NICU length of stay was significantly longer in infants experiencing transport-related complications than in those with safe transport events (16.2 vs. 11.7 days; *p* = 0.04). No deaths were directly attributed to transport-related events.

## 4 Discussion

### 4.1 Principal findings

This prospective pilot study found that (1) 51.4% of intrahospital neonatal transport events were complicated by adverse events under the WHO hypothermia definition, generating 100 adverse events, with 89% occurring on the return leg or post-return period; (2) hypothermia was the dominant event type (74.0%): mild (36.0°C–36.4°C, 40 events) and moderate (32°C–35.9°C, 34 events); (3) moderate, but not mild, hypothermia was independently associated with support escalation (41.2% vs. 18.6%, *p* 0.018); (4) surgical indication was the strongest independent predictor, achieving strong BMA evidence for the secondary outcome (PIP 0.880; OR 4.95, 95% CrI 1.43–17.2); (5) VIS provided no predictive advantage over the vasoactive drug count; and (6) the pretransport SpO_2_/FiO_2_ ratio had clinically useful discrimination for the secondary outcome (AUC 0.735, threshold 372).

### 4.2 Hypothermia: mechanisms, the WHO threshold, and the return-leg risk

Hypothermia was the dominant adverse event type (74.0% of primary AEs). Its temporal pattern was highly informative: all 34 episodes of moderate hypothermia and 34 of 40 episodes of mild hypothermia occurred during the return leg or post-return period, with only six episodes of mild hypothermia occurring during the outward trip. This asymmetry reflects the cumulative thermal burden of transport exposure—neonates lose heat progressively during procedures in operating theaters and imaging suites, and thermal deficits become clinically apparent only when routine monitoring resumes upon NICU readmission (6, 7).

Neonatal thermal loss during intrahospital transport occurs through four simultaneous mechanisms: (1) Radiation—exposure to ambient temperatures in operating theaters and imaging suites (typically 18°C–22°C per international standards), well below the thermoneutral range for neonates; (2) Conduction—direct contact with non-prewarmed transport surfaces (no heated transport mattress was available at this institution during the study period); (3) Convection—air movement during transit through hospital corridors; and (4) Evaporation—particularly pronounced in postoperative neonates with exposed wound sites or preterm infants with immature skin barriers. The cumulative thermal debt model is strongly supported by the temporal pattern observed.

The near-complete absence of incubator use (4.3% of outward trips) in a cohort in which 74% of adverse events are thermal in nature represents the most immediately actionable quality gap. The 19.5 percentage-point difference in unsafe transport rates between the primary (51.4%) and secondary (31.9%) definitions reflects 27 transport events in which mild hypothermia alone occurred—events systematically missed by the <36.0°C threshold and associated in this cohort with no significant escalation of support. The WHO standard of <36.5°C is evidence-based and should be the institutional benchmark for transport safety monitoring.

The CHD subgroup had paradoxically lower adverse event rates, likely reflecting shorter durations and targeted preparation; however, this finding is hypothesis-generating and warrants dedicated prospective investigation in larger cohorts.

Evidence-based thermal protection interventions, in order of priority, are as follows: (1) Routine transport incubator use for all surgical cases and non-surgical transports >60 min; (2) Occlusive polyethylene wrapping for preterm infants, shown to reduce evaporative heat loss by up to 45%; (3) Chemical exothermic mattresses (e.g., Transwarmer) for transports>30 min; (4) Forced-air warming devices (e.g., Bair Hugger) as a postoperative adjunct; (5) An ambient temperature protocol: operating theater and imaging suite ≥26°C during neonatal procedures; and (6) A standardized readmission protocol with mandatory axillary temperature measurement and documentation within 10 min of returning to the NICU as a transport quality audit metric.

### 4.3 Surgical indication: dominant and robust risk predictor

Surgical indication was the only predictor that achieved strong evidence in either analysis. The PIP gradient, which increased from primary (0.616, moderate) to secondary (0.880, strong), quantifies how much of the surgical risk concentrates in clinically significant events beyond mild hypothermia: surgical transports involve postanesthetic hemodynamic and thermoregulatory instability, greater physiological complexity, longer durations, and readmission during a vulnerable recovery phase (6, 12, 19).

Surgical procedure subtype analysis (Supplementary Table S1) revealed esophageal atresia (9/9, 100% primary unsafe), gastroschisis (3/3, 100%), and PDA ligation (4/4, 100%) as the highest-risk elective procedures. Both urgent subgroups—ruptured omphalocele (2/2, 100%) and peritonitis (4/5, 80%)—had the highest overall adverse event rates and longest transport durations. Cardiac catheterization/stenting was the only surgical subgroup with 0% adverse events despite having the longest median duration (219 min), likely reflecting the dedicated preparation of the cardiac team.

In practice, surgical transport should trigger the highest level of preparation: mandatory senior NICU physician attendance, a complete thermal protection checklist, prewarming of the NICU space, and a standardized post-transport reassessment within 30 min of return (7, 20, 21).

### 4.4 VIS as a risk predictor: null finding

VIS achieved negligible BMA evidence in both analyses (PIP 0.083 primary and 0.079 secondary), equivalent to the vasoactive drug count (PIP 0.080). Three factors explain this: (1) the distribution was highly right-skewed with 76.1% VIS = 0, providing minimal information beyond a binary indicator; (2) VIS was nearly perfectly collinear with the vasoactive count (*r* = 0.927); and (3) surgical indication dominated the model space, absorbing variance attributable to hemodynamic severity.

VIS >0 was present in 34.7% of surgical vs. 11.9% of nonsurgical transport events. Pretransport VIS calculation adds no practical value to transport risk stratification in this cohort; vasoactive drug count provides equivalent information with a lower clinical burden.

### 4.5 SpO_2_/FiO_2_ ratio: outcome-specific performance

The SpO_2_/FiO_2_ ratio demonstrated meaningfully superior performance for the secondary outcome (AUC 0.735 vs. 0.644). The dilution in primary outcome performance reflects the inclusion of mild hypothermia events, which are not mechanistically linked to pretransport oxygenation status. At a threshold of 372 for the secondary outcome, the ratio provides clinically actionable triage: infants with SpO_2_/FiO_2_ < 372 should receive the same enhanced preparation as surgical cases. This metric is particularly practical in LMIC settings where arterial blood gases may not be routinely available. Given the pilot nature of this study, the threshold of 372 is hypothesis-generating and requires prospective validation in larger multicenter neonatal cohorts before incorporation into formal transport triage criteria.

### 4.6 The 89% return-leg risk and post-transport reassessment

The concentration of 89% of adverse events on the return leg or in the post-return period indicates that thermal and oxygenation deficits accumulate silently during procedures and manifest clinically upon returning to the NICU, when systematic monitoring resumes. A standardized readmission protocol—axillary temperature and SpO_2_/FiO_2_ assessment within 10 min of return, documented as a mandatory transport quality metric—would capture and accelerate treatment of these events.

### 4.7 Clinical implications

First, the WHO hypothermia threshold (<36.5°C) should be adopted as the institutional standard for transport safety monitoring, with temperature at readmission serving as a mandatory audit metric. Second, thermal protection must be operationalized as a pretransport checklist item: prewarming of transport surfaces, occlusive polyethylene wrapping for preterm infants, incubator use for transports with reliable heating capacity, and ensuring the rapid restoration of optimal thermal conditions upon NICU readmission. Third, surgical indication and SpO_2_/FiO_2_<372 are the two most actionable pretransport risk signals and should trigger enhanced preparation and senior staffing. Fourth, VIS calculation adds no benefit over simply noting whether vasoactive support is present. Fifth, institutions with lower surgical case mix proportions should expect correspondingly lower adverse event rates, and the secondary outcome rate of 31.9% may be more broadly generalizable than the primary rate of 51.4%. Finally, simulation-based training, structured intrahospital transport protocols, and prospective audit frameworks specifically tailored to neonatal patients could reduce variability in practice and sustain safety improvements.

### 4.8 Strengths and limitations

This study has several methodological strengths. It represents one of the first prospective evaluations of intrahospital neonatal transport safety in a tertiary pediatric center in Vietnam, in a high-risk cohort dominated by surgical diagnoses. The event-level analysis captured cumulative risk from multiple transport episodes per infant. Within-infant dependency was low (*r* 0.169, *p* 0.490) and was further examined in a first-event-only sensitivity analysis, confirming the directional robustness of the primary finding. The use of BMA explicitly addressed model uncertainty—a meaningful advantage in pilot studies with modest sample sizes—and yielded robust, well-characterized predictors with quantified posterior probabilities.

Several limitations warrant acknowledgment. The single-center design and 3-month study period window limit generalizability. Ambient temperatures in operating theaters and imaging suites were not systematically recorded, limiting the mechanistic modeling of thermal loss rates. The primary adverse event rate of 51.4% reflects this institution's high surgical case mix (52.2% of transport events), substantially higher than that of general neonatal transport studies (20%–35%). The secondary outcome rate of 31.9%—which excludes mild hypothermia—may be more broadly generalizable, aligning with adverse event rates of 25%–30% reported in non-surgical cohorts from high-income settings. Institutions benchmarking against these findings should apply case-mix adjustment for the proportion of surgical transports.

Validated illness severity scores (CRIB II, SNAP-II) and systematic laboratory data were not collected, which may have underestimated the level of subclinical deterioration, limiting the ability to adjust for baseline physiological vulnerability independent of transport. An important mechanistic distinction exists between two partially overlapping constructs captured by the surgical indication variable: (1) the underlying surgical condition, conferring higher baseline physiological vulnerability independent of transport; and (2) the surgical transport act itself, involving the longest durations and greatest thermal exposure. As this is an observational study, causal inference is not possible, and residual confounding by illness severity remains likely despite multivariable adjustment. The BMA identifies the surgical transport event as the dominant risk signal, but it cannot separate these mechanisms.

Despite prospective data collection, the sample size remains modest for multivariable analyses, and estimates with wide credible intervals indicate the limited precision of the effect estimates, which should be interpreted cautiously. These findings should therefore be considered hypothesis-generating; larger multicenter studies incorporating CRIB II or SNAP-II are needed to validate and disentangle these pathways.

## 5 Conclusions

In this prospective pilot study, the WHO-standard hypothermia threshold revealed that 51.4% of intrahospital neonatal transport events were complicated by adverse events, with 89% occurring on the return leg or post-return period. Hypothermia accounted for 74.0% of events—distributed between mild (36.0°C–36.4°C, 40.0%) and moderate (32°C–35.9°C, 34.0%) severity. Surgical indication was the strongest independent risk factor (BMA PIP 0.880; OR 4.95, 95% CrI 1.43–17.2), while VIS did not improve predictive performance beyond vasoactive drug count. The SpO_2_/FiO_2_ ratio provided clinically meaningful pretransport risk stratification for the secondary outcome (AUC 0.735, threshold 372). Systematic thermal protection—particularly routine incubator use for surgical transport and transport events exceeding 60 min—together with standardized postreturn temperature assessment and enhanced preparation for high-risk surgical cases, are the highest-priority evidence-based interventions. Larger multicenter studies are needed to validate these risk factors, incorporate illness severity scoring, and evaluate targeted safety interventions.

## Data Availability

The raw data supporting the conclusions of this article will be made available by the authors, without undue reservation.
