# Supplementary material for: Adverse events and clinical risk factors during intrahospital transport of critically ill neonates: a prospective pilot study at a tertiary center in Vietnam
Source: Front Pediatr. 2026 Jun 5;14:1850618. doi: 10.3389/fped.2026.1850618 (PMC13279696; doi:10.3389/fped.2026.1850618)
Supplement: Supplementary file 1 [file Supplementaryfile1.docx]

**Supplementary Material**

**Adverse Events and Clinical Risk Factors During Intrahospital Transport of Critically Ill Neonates: A Prospective Pilot Study at a Tertiary Center in Vietnam**

**Supplementary Table S1. Surgical procedure types, urgency, and transport outcomes — 72 surgical transport events**

| **Surgical procedure type** | **n** | **Urgency** | **Median duration (min)** | **Unsafe primary n (%)** | **Unsafe secondary n (%)** |
| --- | --- | --- | --- | --- | --- |
| Esophageal atresia | 9 | Elective | 126 | 9/9 (100%) | 8/9 (89%) |
| Congenital diaphragmatic hernia | 13 | Elective | 174 | 6/13 (46%) | 4/13 (31%) |
| Anorectal anomalies | 8 | Elective | 156 | 5/8 (62%) | 2/8 (25%) |
| Intestinal obstruction / atresia | 7 | Elective | 162 | 5/7 (71%) | 5/7 (71%) |
| Gastroschisis | 3 | Elective | 108 | 3/3 (100%) | 2/3 (67%) |
| Patent ductus arteriosus ligation | 4 | Elective | 153 | 4/4 (100%) | 2/4 (50%) |
| Cardiac catheterization / stent | 4 | Elective | 219 | 0/4 (0%) | 0/4 (0%) |
| Hirschsprung disease | 4 | Elective | 225 | 1/4 (25%) | 1/4 (25%) |
| Retinopathy of prematurity / Avastin therapy | 3 | Elective | 66 | 1/3 (33%) | 1/3 (33%) |
| Neurosurgical (external ventricular drain / ventriculoperitoneal shunt) | 2 | Elective | 108 | 2/2 (100%) | 2/2 (100%) |
| Omphalocele (ruptured) | 2 | Urgent | 129 | 2/2 (100%) | 2/2 (100%) |
| Peritonitis / perforation | 5 | Urgent | 222 | 4/5 (80%) | 4/5 (80%) |
| Other (lymphangioma, teratoma, airway, splenic rupture) | 8 | Mixed | 168 | 5/8 (62%) | 3/8 (38%) |
| **Total** | **72** | **8 urgent** | **162** | **47/72 (65.3%)** | **36/72 (50.0%)** |

Abbreviations: PDA, patent ductus arteriosus; ROP, retinopathy of prematurity; ECMO, extracorporeal membrane oxygenation. No patient required ECMO during transport. Unsafe primary was defined as at least one adverse event according to the WHO-based primary definition, including hypothermia <36.5°C or another clinically significant event. Unsafe secondary was defined as at least one event according to the secondary definition, including moderate hypothermia <36.0°C or another clinically significant event. Median duration is shown for each procedure group.

**Supplementary Table S2. Congenital heart disease (CHD) vs. non-CHD transport events — subgroup analysis**

| **Characteristic** | **CHD (n = 23 transport events)** | **Non-CHD (n = 115 transport events)** |
| --- | --- | --- |
| Unsafe primary, n (%) | 7 (30.4%) | 64 (55.7%)* |
| Unsafe secondary, n (%) | 3 (13.0%) | 41 (35.7%)* |
| Any hypothermia (<36.5°C), n (%) | 6 (26.1%) | 62 (53.9%) |
| Invasive mechanical ventilation, n (%) | 14 (60.9%) | 77 (67.0%) |
| VIS > 0, n (%) | 9 (39.1%) | 24 (20.9%) |
| Median transport duration (min) | 60 | 96 |
| Surgical indication, n (%) | 11 (47.8%) | 61 (53.0%) |

*p < 0.05 (Fisher’s exact test). Abbreviations: CHD, congenital heart disease; MV, mechanical ventilation; VIS, vasoactive-inotropic score. The paradoxically lower adverse event rate in CHD likely reflects shorter median transport duration (60 vs. 96 min), 0% adverse events in cardiac catheterization/stent procedures (n = 4, median duration 219 min), and targeted cardiac NICU team preparation. This analysis is hypothesis-generating given the small subgroup size.
